# ESCRT requirements for EIAV budding

**DOI:** 10.1186/1742-4690-10-104

**Published:** 2013-10-09

**Authors:** Virginie Sandrin, Wesley I Sundquist

**Affiliations:** 1 Department of Biochemistry, University of Utah School of Medicine, Salt Lake City 84112-5650, Utah, USA

**Keywords:** Virus budding, Gag, ALIX, CHMP4, CHMP2, VPS4

## Abstract

**Background:**

Retroviruses and many other enveloped viruses usurp the cellular ESCRT pathway to bud from cells. However, the stepwise process of ESCRT-mediated virus budding can be challenging to analyze in retroviruses like HIV-1 that recruit multiple different ESCRT factors to initiate budding.

**Results:**

In this study, we characterized the ESCRT factor requirements for budding of Equine Infectious Anemia Virus (EIAV), whose only known direct ESCRT protein interaction is with ALIX. siRNA depletion of endogenous ESCRT proteins and “rescue” experiments with exogenous siRNA-resistant wild type and mutant constructs revealed budding requirements for the following ESCRT proteins: ALIX, CHMP4B, CHMP2A and VPS4A or VPS4B. EIAV budding was inhibited by point mutations that abrogate the direct interactions between ALIX:CHMP4B, CHMP4B:CHMP2A, and CHMP2A:VPS4A/B, indicating that each of these interactions is required for EIAV budding. Unexpectedly, CHMP4B depletion led to formation of multi-lobed and long tubular EIAV virions.

**Conclusions:**

We conclude that EIAV budding requires an ESCRT protein network that comprises EIAV Gag-ALIX-CHMP4B-CHMP2A-VPS4 interactions. Our experiments also suggest that CHMP4B recruitment/polymerization helps control Gag polymerization and/or processing to ensure that ESCRT factor assembly and membrane fission occur at the proper stage of virion assembly. These studies help establish EIAV as a streamlined model system for dissecting the stepwise processes of lentivirus assembly and ESCRT-mediated budding.

## Background

Many enveloped viruses usurp the cellular ESCRT pathway to bud from cells. The ESCRT pathway also mediates membrane fission during vesicle formation at the multivesicular body (MVB), abscission of the intercellular bridge, and shedding microvesicle formation at the plasma membrane (reviewed in [1-8]). All of these processes require membranes to be constricted *toward* the cytoplasm, ultimately resulting in membrane fission. The ESCRT pathway is therefore mobile machinery that is targeted to different cellular membranes to mediate different “reverse topology” membrane fission events.

ESCRT factors assemble in a stepwise fashion in which “early-acting” factors bind site-specific adaptors and then recruit the “late-acting” factors that mediate membrane fission and ESCRT factor recycling. The ESCRT pathway was first identified through genetic studies of MVB sorting in *S. cerevisiae*[8-13], and this system continues to serve as the paradigm for understanding ESCRT protein assembly and function. Attractive features of the yeast system include robust MVB protein sorting assays [14], the ability to make genetic knockouts, and the relative simplicity of the yeast ESCRT machinery, which often contains single versions of proteins that have diverged into multi-protein families in mammals. Even *S. cerevisiae* has more than 20 ESCRT-associated proteins, however, and it has therefore proven useful to divide them into essential factors that are required for MVB sorting, and non-essential accessory factors that appear to modulate or regulate core protein functions (reviewed in reference [8]). Although these distinctions are not absolute, the five consensus core components of the *S. cerevisiae* MVB sorting pathway are: 1) ESCRT-0 (a two protein complex), 2) ESCRT-I (a four protein complex), 3) ESCRT-II (a three protein complex), 4) Vps20p/*CHMP6*, Snf7p/*CHMP4A/B/C*, Vps24p/Did3p/*CHMP3*, and Vps2p/Did4p/*CHMP2A/B* (the core ESCRT-III factors, with human homolog names in italics), and 5) Vps4p/*VPS4A/B*.

The core ESCRT factors are recruited sequentially to sites of *S. cerevisiae* MVB protein sorting [8]. The ESCRT-0 adaptor initially concentrates ubiquitylated cargoes on endosomal membranes and recruits the ESCRT-I complex through a direct interaction with the Vps23p/*TSG101* subunit [15]. ESCRT-I recruits ESCRT-II, and the ESCRT-I-II supercomplex helps stabilize the highly curved membrane neck of the emerging vesicle [16,17]. ESCRT-II binds two copies of Vps20p/*CHMP6*, leading to recruitment of the ESCRT-III subunits through direct, ordered interactions between Vps20p/*CHMP6*, Snf7p/*CHMP4A-C*, Vps24p/Did3p/*CHMP3*, and Vps2p/Did4p/*CHMP2A-B*[18-21]. The ESCRT-III subunits appear to form paired helical filaments that constrict the bud neck [19,22-31], although their precise architecture and constriction mechanism are not yet well understood. The polymerized ESCRT-III subunits, particularly Vps2p/*CHMP2A/B* and Snf7p/*CHMP4A/B/C*, also recruit the Vps4p/*VPS4A/B* AAA ATPase, using two different types of C-terminal tail motifs (called MIM-1 and MIM-2 elements) to bind Vps4 MIT domains [32-35]. Vps4p forms hexameric rings, and uses the energy of ATP hydrolysis to remodel the ESCRT-III filaments [22,36,37], resulting in membrane fission and ESCRT-III subunit disassembly and recycling to the cytoplasm. Accessory ESCRT proteins in *S. cerevisiae* include three ESCRT-III-like proteins: Vps44p/*CHMP1A/B*, Ist1p/*IST1*, and Vps60p/*CHMP5*, which interact with both core ESCRT-III subunits and with Vps4p [38-42]; Vta1p/*LIP5*, which binds both Vps60p/*CHMP5*[43,44] and Vps4p/*VPS4A/B*, and stimulates enzyme assembly and ATPase activity [45-48]; and the ESCRT-III adaptor protein, Bro1p/*ALIX*, which binds and stabilizes the Snf7p/*CHMP4A/B/C* filaments, and recruits the deubiquitinating enzyme, Doa4p/*UBPY*[23,49].

Although the core yeast ESCRT components and their mechanistic functions are largely conserved across eukaryotes, there are likely to be important differences in the way the pathway is used to perform distinct membrane fission reactions, particularly in processes like enveloped virus budding that do not occur in yeast. ESCRT-mediated enveloped virus budding has been most intensively studied for retroviruses, particularly HIV-1 (reviewed in [6,50-53]). The structural Gag proteins of retroviruses initiate ESCRT factor recruitment using one of three well-characterized peptide motifs, termed “late assembly domains”. “P(S/T)AP” late assembly domains function by binding the TSG101 subunit of ESCRT-I; “YP(X)_n_L” late assembly domains function by binding ALIX; and “PPXY” late assembly domains function by binding members of the ESCRT-associated NEDD4 family of ubiquitin E3 ligases. Recent studies, particularly of HIV-1, make it clear that these initial interactions ultimately result in the recruitment of downstream ESCRT-III and VPS4 proteins, which carry out the membrane fission step [54-58]. However, the precise set of downstream factors and protein-protein interactions required for ESCRT-mediated virus budding have not yet been defined unambiguously for any enveloped virus.

One challenge in dissecting how the ESCRT pathway functions in retrovirus budding is that mammalian cells express a large number of isoforms of the different classes of ESCRT factors, including at least 12 distinct subunits of the ESCRT-III family. Adding to this complexity is the fact that the Gag proteins from many retroviruses contain multiple late assembly domains that can bind and recruit different early-acting ESCRT factors. For example, HIV-1 Gag contains both P(S/T)AP and YPX_n_L motifs that bind directly to TSG101/ESCRT-I and ALIX, respectively [59-62]. These two early-acting ESCRT factors can function independently and redundantly, at least in some contexts [63,64], and this redundancy makes it challenging to evaluate the functional requirements for different downstream ESCRT proteins and their interactions. For example, the requirement for ESCRT-II in HIV-1 assembly is controversial, with several groups arguing that the complex is important [56,65], and several others arguing it is not [57,66,67]. In contrast, the Gag protein of the Equine Infectious Anemia Viruses (EIAV) lacks a TSG101/ESCRT-I binding site and is only known to connect to the ESCRT pathway via ALIX [39,40,62,63,68-72]. This apparent simplicity makes EIAV an attractive model system for studying how the ESCRT pathway functions in virus budding. Similarly, the Gag proteins of some SIV strains also bind ALIX but lack identifiable TSG101/ESCRT-I binding sites [73,74], indicating that EIAV can also serve as a paradigm for the budding of this class of primate lentiviruses. Other attractive aspects of the EIAV system include the availability of: 1) EIAV-based reporter vectors [75,76], and 2) analyses of the temporal recruitment of fluorescent ESCRT factors to assembling EIAV Gag particles [54]. The functional requirements for late-acting ESCRT factors in EIAV budding have not yet been tested, however. We therefore examined the requirements for core ESCRT factors in EIAV budding, with the ultimate goal of developing EIAV as a useful model system for characterizing how different ESCRT factors function in lentivirus budding.

## Results

### EIAV release requires ALIX, CHMP2, CHMP4 and VPS4 proteins

We used siRNA depletion experiments to test the requirements for all of the human ESCRT factors that correspond to core ESCRT factors for MVB sorting in *S. cerevisiae*[8]: ESCRT-I, ESCRT-II, CHMP6, CHMP4A-C, CHMP3, CHMP2A-B, and VPS4A-B, plus ALIX. Preliminary surveys, described in greater detail in Additional file 1 and shown in Additional file 2: Figure S1 and Additional file 3: Figure S2, demonstrated that: 1) CHMP2A is the primary human CHMP2 isoform that contributes to EIAV budding from 293T cells (Additional file 2: Figure S1A). Co-depletion of CHMP2A and CHMP2B further enhances inhibition of EAIV release, and both CHMP2 proteins were therefore co-depleted in subsequent analyses of CHMP2 function. 2) CHMP4B is the primary human CHMP4 isoform that contributes to EIAV budding (Additional file 2: Figure S1B). CHMP4C depletion did not significantly inhibit EIAV budding under any conditions tested, whereas co-depletion of CHMP4A with CHMP4B enhanced inhibition. CHMP4A/B co-depletion was therefore used in subsequent tests for CHMP4 function. 3) Depletion of EAP20 (ESCRT-II), CHMP6 (ESCRT-III) or CHMP3 (ESCRT-III) had no measurable effect on EIAV release or infectivity (Additional file 3: Figure S2), and these factors therefore were not studied further.

**Figure 1:**
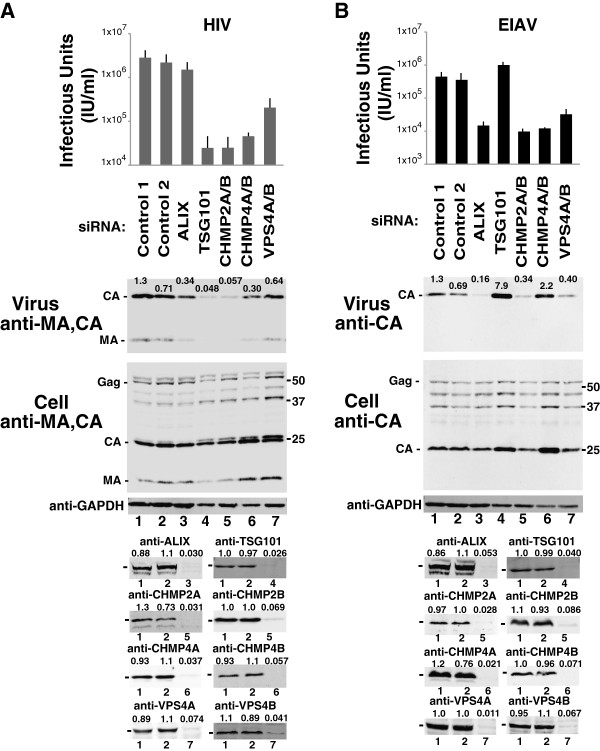
**ESCRT requirements for HIV-1 and EIAV release and infectivity.** Viral titers (top panel, note log scale), and western blots showing levels of virion-associated Gag proteins (panel 2, “Virus”) and the designated intracellular viral and cellular proteins in 293T cells (lower panels, “Cell”) expressing HIV-1 **(A)** or EIAV **(B)**, with positions of molecular weight markers shown at right. Virus-producing cells were treated with control siRNAs (lanes 1 and 2) or siRNAs that depleted ALIX (lane 3), TSG101 (lane 4), CHMP2A and CHMP2B (lane 5), CHMP4A and CHMP4B (lane 6), or VPS4A and VPS4B (lane 7). Bottom panels show GAPDH loading controls and cellular levels of the designated ESCRT protein following treatment with the control siRNA (equivalent to control lanes 1 and 2) or a specific siRNA (equivalent to the lane designated beneath the blot). Band intensities were integrated for the shown blots to quantify levels of virus release (panel 2, CA band intensities) and target protein depletion (bottom panels), and values are relative to the average intensities of the two control lanes. Error bars in the top panel show the standard deviation from the mean of 3–6 independent repetitions of the experiment.

Requirements for the remaining core ESCRT proteins in HIV-1 and EIAV release and infectivity are compared in Figure 1A and B, respectively. For these experiments, 293T cells were transfected with expression constructs for either HIV-1_NL4-3_ or EIAV, together with either control siRNAs (lanes 1 and 2, denoted “Control 1 and 2”), or with siRNAs that targeted ALIX (lane 3), TSG101 (lane 4), CHMP2A and B (lane 5), CHMP4A and B (lane 6), or VPS4A and B (lane 7). Viral titers were measured in single-cycle infectivity assays (panel 1), and virion release was analyzed using western blotting to quantify the levels of virion-associated CA proteins released into the culture supernatant (panel 2, “Virus”). Western blots of soluble cell extracts were also performed to visualize Gag protein expression and processing (panel 3), GAPDH levels (“Control”, panel 4), and the efficiency of target protein depletion (bottom panels). In every case, the siRNA treatment reduced target protein levels more than 10-fold.

As expected, our results for the HIV-1 control (Figure 1A) agree well with previous reports [57,59,62,77]. Depletion of ALIX modestly reduced virion release and infectivity (compare lane 3 to lanes 1 and 2, 3-fold reduction in virion release and 2-fold reduction in infectivity), whereas greater reductions were seen upon depletion of TSG101 (lane 4, 21-fold reduction in virion release and 101-fold reduction in infectivity), CHMP2A/B (lane 5, 18- and 101-fold reductions, respectively), CHMP4A/B (lane 6, 3- and 55-fold reductions, respectively) and VPS4A/B (lane 7, 2- and 12-fold reductions, respectively). These experiments confirm that TSG101, CHMP2, CHMP4 and VPS4 proteins all make important contributions to HIV-1 release from 293T cells, and that ALIX makes a modest, but measurable contribution.

As shown in Figure 1B, the EIAV requirements for early-acting ESCRT factors differed from those of HIV-1 because ALIX was more important for EIAV whereas TSG101 was unimportant. ALIX depletion reduced EIAV release and infectivity by 6-fold and 27-fold, respectively (compare lane 3 to lanes 1 and 2), whereas TSG101 depletion actually *increased* virion release and infectivity modestly (lane 4, 8- and 2-fold increases, respectively). These results are consistent with previous reports that the EIAV p9^Gag^ polypeptide contains a functional YPDL late domain that recruits ALIX, but lacks a TSG101 binding site [39,40,62,63,68-72]. We speculate that the modest increases in virion release and infectivity observed upon TSG101 depletion may reflect competition for late-acting ESCRT factors between EIAV budding and other cellular processes, which is relieved when TSG101 is depleted.

HIV-1 and EIAV generally exhibited similar requirements for late-acting ESCRT-III and VPS4 factors, albeit with several notable exceptions. Like HIV-1, EIAV infectivity was strongly reduced upon CHMP2A/B and CHMP4A/B depletion (Figure 1B, lanes 5 and 6, 41- and 33-fold infectivity reductions, respectively), and moderately reduced upon VPS4A/B depletion (lane 7, 12-fold infectivity reduction). The two reproducible differences between HIV-1 and EIAV were: 1) EIAV appears to rely on CHMP2A more than HIV-1 does (where single-protein depletions of both CHMP2A and CHMP2B produced measurable titer reductions) (Additional file 2: Figure S1A and ref. [57]). 2) CHMP4B depletion did not reduce EIAV Gag release, despite the infectivity reductions. Indeed, levels of virion-associated EIAV CA^Gag^ reproducibly *increased* when CHMP4B was depleted, either alone or in conjunction with other CHMP4 proteins (e.g., see Figure 1B, panel 2, compare lane 6 to lanes 1 and 2 and Additional file 2: Figure S1B, panel 2, compare lanes 4, 6, 8 and 9 to lane 1). The magnitude of the increase varied, ranging from 2-fold (Figure 1B, lane 6) to 19-fold (Additional file 2: Figure S1B, lane 6). This observation suggested that CHMP4B depletion might alter the properties of EIAV virions, and this phenomenon was investigated further using electron microscopy, as described below in the final Results section.

### EIAV release requires an interaction between ALIX and CHMP4B

**Figure 2:**
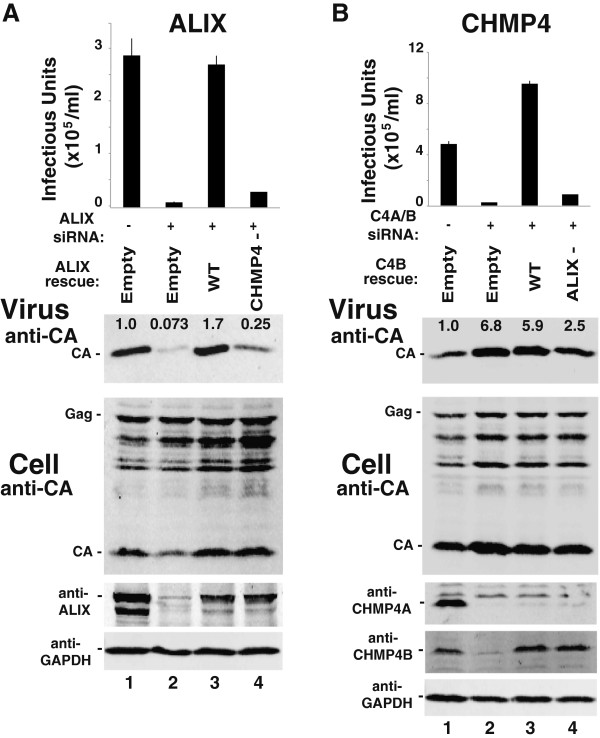
**EIAV release requires an interaction between ALIX and CHMP4B.** Effects on EIAV release and infectivity of ALIX depletion/re-expression **(A)** and CHMP4B depletion/re-expression **(B)**. Viral titers (top panel), and western blots showing levels of virion-associated Gag proteins (panel 2, “Virus”), and the designated intracellular viral and cellular proteins in 293T cells expressing EIAV (lower panels, “Cell”). **(A)** EIAV producer cells were transfected with a control siRNA (lane 1) or an siRNA that depleted ALIX (lanes 2–4), together with an empty vector control (lanes 1 and 2), or with a vector expressing either siRNA-resistant wild type ALIX (lane 3) or an ALIX protein with a mutation that impairs CHMP4B binding (ALIX_I212D_, “CHMP4-”, lane 4). **(B)** EIAV producer cells were transfected with a control siRNA (lane 1) or siRNAs that depleted CHMP4A/B (lanes 2–4), together with an empty vector control (lanes 1 and 2), or with a vector expressing siRNA-resistant wild type CHMP4B (lane 3) or a CHMP4B protein with mutations that impair ALIX binding (CHMP4B_L217A,W220A_, denoted “ALIX-”, lane 4). Other panels are equivalent to the corresponding panels in Figure 1. Error bars in the top panels show the range from the mean of two independent repetitions of the experiments, performed in parallel.

Functional rescue experiments were performed using siRNA-resistant constructs to re-express exogenous ESCRT proteins following depletion of their endogenous counterparts. These experiments confirmed the specificity of the siRNA depletion phenotypes, and were also used to test the functional effects of ESCRT protein mutations. As shown in Figure 2A, the strong detrimental effects of ALIX depletion on EIAV release and infectivity could be rescued fully by overexpression of exogenous ALIX from an siRNA resistant construct (compare lane 3 to lanes 1 and 2). In contrast, an ALIX mutation that impaired CHMP4 binding also impaired EIAV release and infectivity (ALIX (I212D), denoted “CHMP4-”, compare lanes 4 and 3, and see ref. [63]), even though the wild type and mutant proteins were expressed at comparable levels (panel 4).

Similar effects were seen for an inactivating mutation on the other side of the ALIX-CHMP4B interface. As shown in Figure 2B, the inhibition of infectious EIAV particle release caused by co-depletion of CHMP4A and CHMP4B could be fully rescued by re-expression of wild type CHMP4B from an siRNA construct (compare lane 3 to lanes 1 and 2), but not by a mutant CHMP4B protein that could not bind ALIX (CHMP4B (L217A,W220A), denoted “ALIX-”, compare lane 4 to lane 3, and see ref. [57]). These results imply that ALIX and CHMP4B must interact directly to support release of infectious EIAV.

### The CHMP2-CHMP4 interaction contributes to EIAV release

**Figure 3:**
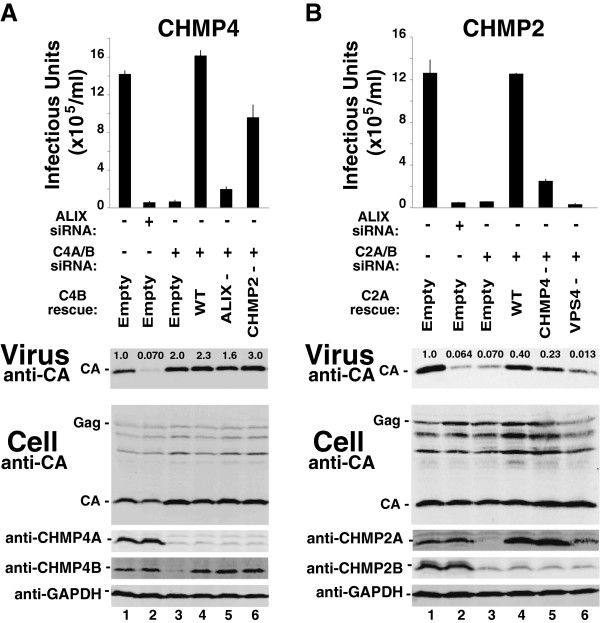
**CHMP2-CHMP4 and CHMP2-VPS4 interactions contribute to EIAV release.** Effects on EIAV release and infectivity of CHMP4 depletion/re-expression **(A)** and CHMP2 depletion/re-expression **(B)**. Viral titers (top panel), and western blots showing levels of virion-associated Gag proteins (panel 2, “Virus”), and the designated intracellular viral and cellular proteins in 293T cells expressing EIAV (lower panels “Cell”). **(A)** EIAV producer cells were transfected with a control siRNA (lane 1), an siRNA that depleted ALIX (lane 2, positive control) or siRNAs that depleted CHMP4A and CHMP4B (lanes 3–6), together with an empty vector control (lanes 1–3), or with vectors expressing either siRNA-resistant wild type CHMP4B (lane 4) or CHMP4B proteins with mutations that impair binding to ALIX (CHMP4B_L217A,W220A_, denoted “ALIX-”, lane 5) or CHMP2A (CHMP4B_104EVLK107/AAAA_, denoted “CHMP2-”, lane 6). **(B)** EIAV producer cells were transfected with a control siRNA (lane 1), an siRNA that depleted ALIX (lane 2, positive control) or siRNAs that depleted CHMP2A and CHMP2B (lanes 3–6), together with an empty vector control (lanes 1–3), or with vectors expressing either siRNA-resistant wild type CHMP2A (lane 4) or CHMP2A proteins with mutations that impair binding to CHMP4B (CHMP2A_R24A,R27A,R31A_, denoted “CHMP4-”, lane 5) or VPS4 (CHMP2A_L216D,L219D_, denoted “VPS4-”, lane 6). Other panels are equivalent to the corresponding panels in Figure 1. Error bars in the top panel show the range from the mean of two independent repetitions of the experiment, performed in parallel.

Analogous experiments were used to test the functional requirements for CHMP4 and CHMP2 in EIAV release (Figure 3). As shown in Figure 3A, an exogenously expressed wild type CHMP4B protein fully rescued viral infectivity (compare lanes 3 and 4), whereas a mutant CHMP4B protein that was impaired for CHMP2 binding rescued EIAV infectivity only partially (CHMP4B (_104_EVLK_107_ to _104_AAAA_107_), denoted “CHMP2-”, compare lanes 6 and 4, and see ref. [57]). Similarly, an exogenously expressed wild type CHMP2A protein rescued the defects in EIAV budding induced by depletion of CHMP2A and CHMP2B (Figure 3B, compare lanes 4 and 3), whereas a mutant CHMP2A protein impaired in CHMP4 binding rescued poorly (CHMP2A (R24A,R27A,R31A), denoted “CHMP4-”, compare lanes 5 and 4, and see ref. [57]). These experiments indicate that CHMP4B and CHMP2A interact directly during the process of EIAV budding. The detrimental interaction mutations did not completely inhibit EIAV budding, however, possibly because CHMP3 can also bind and help bridge these two proteins [30,56].

### EIAV release requires VPS4 ATP, MIM1 and MIM2 binding activities

**Figure 4:**
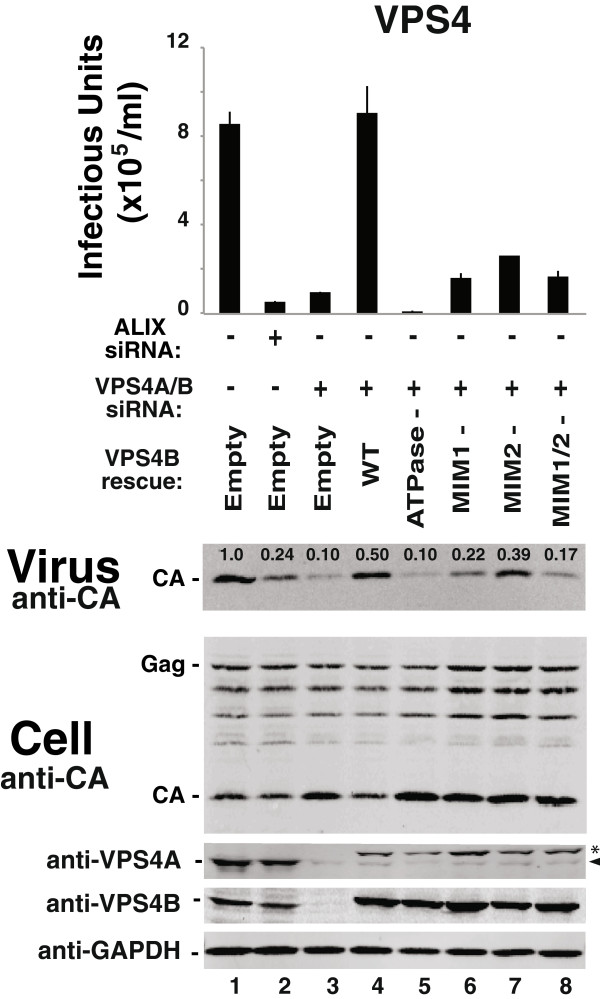
**EIAV release requires VPS4 ATP, MIM1, and MIM2 binding activities.** Effects on EIAV release and infectivity of VPS4 depletion/re-expression. Viral titers (top panel), and western blots showing levels of virion-associated Gag proteins (panel 2, “Virus”), and the designated intracellular viral and cellular proteins in 293T cells expressing EIAV (lower panels “Cell”). EIAV producer cells were transfected with a control siRNA (lane 1), an siRNA that depleted ALIX (lane 2, positive control) or siRNAs that depleted VPS4A and VPS4B (lanes 3–8), together with an empty vector control (lanes 1–3), or with vectors expressing either siRNA-resistant wild type VPS4B (lane 4) or VPS4B proteins with inactivating mutations within the ATPase binding site (VPS4B_K180Q_, denoted “ATPase-”, lane 5), the MIM1 binding site (VPS4B_L66D_, denoted “MIM1-”, lane 6), the MIM2 binding site (VPS4B_A15D_, denoted “MIM2-”, lane 7), or both MIM1 and MIM2 binding sites (VPS4B_L66D,A15D_, denoted “MIM1/2-”, lane 8). Other panels are equivalent to the corresponding panels in Figure 1. The arrow and asterisk in the anti-VPS4A panel designate bands that correspond to endogenous VPS4A and a band that arises owing to antibody cross-reactivity with overexpressed VPS4B, respectively. Error bars in the top panel show the range from the mean of two independent repetitions of the experiment, performed in parallel.

The VPS4 protein requirements for EIAV release were also tested using functional rescue experiments. As shown in Figure 3B, a CHMP2A protein with point mutations in the terminal MIM1 element that inhibit VPS4 MIT binding was unable to rescue virus budding (CHMP2A (L216D,L219D), denoted “VPS4-”, compare lanes 4 and 6, and see refs. [33,35,57]). This result indicates that CHMP2A must bind VPS4 during EIAV budding. Similar effects were also seen for an inactivating mutation on the other side of the CHMP2-VPS4 interface. As shown in Figure 4, the wild type VPS4B protein completely rescued the defect in EIAV infectivity induced by co-depletion of endogenous VPS4A and VPS4B (compare lanes 4 and 3), whereas a VPS4B protein with an inactivating point mutation in the MIM1 binding site did not rescue viral infectivity significantly (VPS4B (L66D), denoted “MIM1-”, compare lanes 6 and 4, and see refs. [33-35,57]).

VPS4 proteins can bind ESCRT-III substrates through a second type of MIT-binding motif (termed MIM2) [34,78]. This activity was also apparently required for efficient rescue of EIAV budding and infectivity because a VPS4B protein with an inactivating mutation in the MIM2 binding site rescued EIAV release and infectivity only slightly (VPS4B (A15D), denoted “MIM2-”, compare lanes 7 and 4, and see [34]). Similarly, the VPS4 ATPase activity was required because a mutant VPS4B protein that could not bind ATP failed to rescue EIAV budding (VPS4B (K180Q), denoted “ATPase-” compare lanes 5 and 4, and see refs. [37,39,59,79]). Expression of the VPS4B ATPase-defective mutant decreased EIAV infectivity to an even greater extent than did depletion of the endogenous VPS4 proteins alone, consistent with previous reports that this VPS4B construct is a potent dominant negative inhibitor of EIAV release [72,80].

### CHMP4 recruitment helps terminate EIAV Gag polymerization

**Figure 5:**
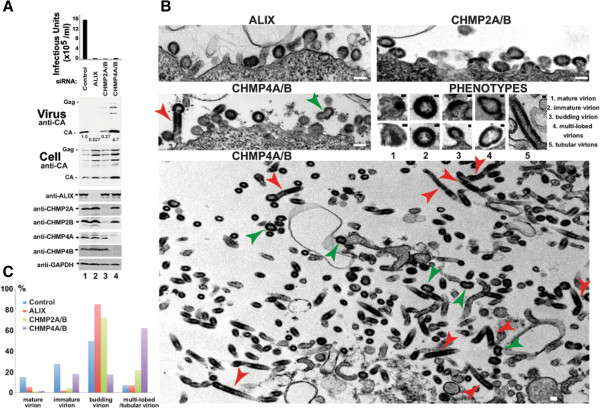
**EM analyses of EIAV budding defects in the absence of ALIX, CHMP2A/B and CHMP4A/B proteins. (A)** EIAV infectivity and release in control cells (lane 1) or cells depleted of ALIX, CHMP2, or CHMP4 proteins (lanes 2–4, respectively). Panels show viral titers (top panel), and western blots showing levels of virion-associated Gag proteins (panel 2, “Virus”) and the designated intracellular viral and cellular proteins in 293T cells expressing EIAV (lower panels, “Cell”). Virus-producing cells were treated with control siRNAs (lane 1) or siRNAs that depleted ALIX (lane 2), CHMP2A and CHMP2B (lane 3), or CHMP4A and CHMP4B (lane 4). Bottom panels show cellular levels of the designated ESCRT protein following treatment with the different siRNAs. Integrated band intensities are shown for virus release (panel 2, CA band intensities for the shown blot, relative to the control lane). **(B)** Thin section transmission EM images showing: cell-associated EIAV virions from cells depleted of ALIX, CHMP2 or CHMP4 proteins (panels 1–3, respectively), representative examples of the different virion phenotypes (panel 4), and an expanded view showing the phenotypes of virions associated with cells depleted of CHMP4 proteins (panel 5). Green and red arrows in panels 3 and 5 highlight virions with a multi-lobed or tubular phenotypes, respectively. Scale bars are 100 nm (white bars) or 20 nm (black bars). TEM images of EIAV virions released from control cells are shown in Additional file 4: Figure S3. **(C)** Relative frequencies (percentage) of the different phenotypes seen for cell-associated EIAV virions associated with control cells (blue) or cells depleted of ALIX, CHMP2A/B, or CHMP4A/B (red, green and purple, respectively).

As noted above, CHMP4B was necessary for EIAV infectivity, but the release of virion-associated EIAV Gag was reproducibly elevated in cells that lacked CHMP4B. This effect is again evident in Figure 5A, where co-depletion of CHMP4A and CHMP4B *reduced* the viral titer 30-fold, but *increased* virion-associated Gag protein levels 4.7-fold (panel 2, compare lanes 1 and 4). Gag processing was also less complete in virions released from cells lacking CHMP4 proteins as compared to the control case. To characterize these phenomena further, we used transmission electron microscopy (EM) to visualize the morphology of the cell-associated EIAV virions produced from control 293T cells, or from cells depleted of ALIX, CHMP2 or CHMP4 proteins (Figure 5B and Additional file 4: Figure S3). These experiments revealed that cells lacking CHMP4A/B produced large numbers of highly aberrant virions that were either multi-lobed and/or tubular (Figure 5B, bottom panel, highlighted with green and red arrows, respectively), as well as immature EIAV virions that were budding and/or closely associated with the cell surface in the imaged sections (Figure 5B, left middle panel). Although some of these particles were associated with the plasma membrane (left middle panel), many were distant from any cell surface in the imaged sections (bottom panel). The tubular EIAV virions were very long, often hundreds of nanometers in length, even within the plane of a single 80–100 nm section (red arrows). In contrast, cells lacking ALIX and CHMP2A/B produced many fewer aberrant particles, and instead exhibited a more traditional “late assembly” phenotype in which immature virions typically remained tethered to the cell surface through unresolved membrane “necks” (Figure 5B, top two panels).

These different virion assembly phenotypes were quantified for a total of 28 virion-producing cells from two independent experiments (Figure 5C). Cell-associated virions were relatively rare in the control case, and we had to examine 1020 total cell sections to find 28 cells that produced 198 identifiable virions. Cell-associated virions were more prevalent in the other cases, where 28 virion-producing cells were identified from: 1282 slices of ALIX-depleted cells that produced 365 scored virions; 927 cell slices of CHMP2A/B-depleted cells that produced 491 virions, and 428 CHMP4A/B-depleted cell slices that produced 1172 virions. Hence, cell-associated viral particles were more prevalent when cells lacked ESCRT factors, consistent with an arrest in virus budding.

The different phenotypes were quantified by counting the following types of cell-associated virions: 1) spherical, mature, cell-free virions, 2) spherical, immature, cell-free virions, 3) spherical budding virions, and 4) multi-lobed or tubular virions (examples of each phenotype are provided in Figure 5B, middle right panel). As expected, EIAV virions produced from control cells were distributed primarily between mature (15%), immature (28%) and budding particles (50%), and multi-lobed/tubular particles were rare (7%). In contrast, the majority of EIAV particles produced in the absence of CHMP4A/B were multi-lobed or tubular (62%). Even this elevated value likely underestimates the true percentage of tubular virions because some were probably incorrectly scored as immature virions when the plane of section was perpendicular to the tube axis. As expected, EIAV virions produced from cells lacking ALIX exhibited more traditional late assembly defects, with the vast majority (85%) remaining connected to the plasma membrane via membrane stalks, and no elevation in multi-lobed/tubular particles. EIAV virions produced from cells lacking CHMP2A/B exhibited a modestly elevated percentage of multi-lobed/tubular virions (22% vs. 7% in the control). Nevertheless, the CHMP2A/B depletion phenotype most closely resembled the ALIX depletion phenotype because most of the observable virions were (arrested) in the process of budding (72%).

These EM data provide an explanation for the apparent discrepancy between measurements of viral titers and virion release (see Figure 5A, panels 1 and 2, which were performed on the same samples as those used for EM analyses). Our interpretation is that some of the highly aberrant multi-lobed/tubular virions containing high levels of Gag proteins may ultimately bud (or break off) from cells that lack CHMP4A/B. These aberrant virions are likely poorly infectious, however, which explains why virion-associated Gag release appears high (but variable), whereas viral titers are consistently low. In contrast, ALIX depletion induces a more traditional “late” assembly phenotype in which immature particles arrest during budding, leading to strong reductions in both virion release (37-fold) and titers (34-fold). Virions produced from cells lacking CHMP2A/B exhibited intermediate phenotypes in both the EM analyses (Figure 5B and C) and in the virion release/infectivity assays, where the dramatic reduction in viral titer (49-fold) was accompanied by only a modest reduction in virion release (4-fold). Hence, depletion of ALIX, CHMP2A/B and CHMP4A/B proteins all induced virus budding defects, but resulted in different phenotypes.

## Discussion

We investigated the core ESCRT factor requirements for EIAV budding, and found that the greatest reductions in EIAV infectivity occurred upon depletion of the single ESCRT factors ALIX, CHMP4B, and CHMP2A (Figures 1B and 2A, and Additional file 2: Figure S1). In each case, EIAV infectivity was reduced at least 4-fold, and viral infectivity was fully rescued upon re-expression of the wild type protein (Figures 2 and 3). Thus, these three factors perform essential, and largely non-redundant roles in EIAV budding. Co-depletion of VPS4A and VPS4B also inhibited EIAV release (Figures 1 and 4), and this defect could be fully rescued by VPS4B alone (Figure 4). Thus, the virus also requires VPS4 activity and VPS4B can meet this requirement. Finally, synergistic effects were observed upon co-depletion of CHMP2A/B and CHMP4A/B (Additional file 2: Figure S1), implying that CHMP2B and CHMP4A may also contribute to EIAV budding, at least when CHMP2A and CHMP4B levels are low.

Single protein depletions of TSG101, EAP20, CHMP2B, CHMP3, CHMP4A, CHMP4C and CHMP6 had little or no effect on EIAV infectivity (Figure 1B and Additional file 2: Figure S1 and Additional file 3: Figure S2), suggesting that none of these proteins perform essential, non-redundant functions. These negative siRNA results must be interpreted with some caution, however, owing to the possibility that small reductions in the kinetics of virus release may have eluded detection in our “end point” virus release and infectivity assays and/or that residual levels of the depleted target proteins were sufficient to retain function. Nevertheless, all target protein levels were reduced at least 8-fold, and in many cases were reduced to nearly undetectable levels.

Our experiments confirm that HIV-1 and EIAV differ in their requirements for TSG101, consistent with the lack of a known TSG101 binding site in EIAV p9^Gag^. The ESCRT-I independence of EIAV is also consistent with the lack of a requirement for EAP20 (an essential component of ESCRT-II, an ESCRT-I binding complex) or CHMP6 (the only ESCRT-III protein known to bind ESCRT-II). Hence, EIAV, and presumably also some SIV strains [73,74], use a streamlined ESCRT-based budding pathway that does not include ESCRT-I, and possibly also other ESCRT factors used by HIV-1.

**Figure 6:**
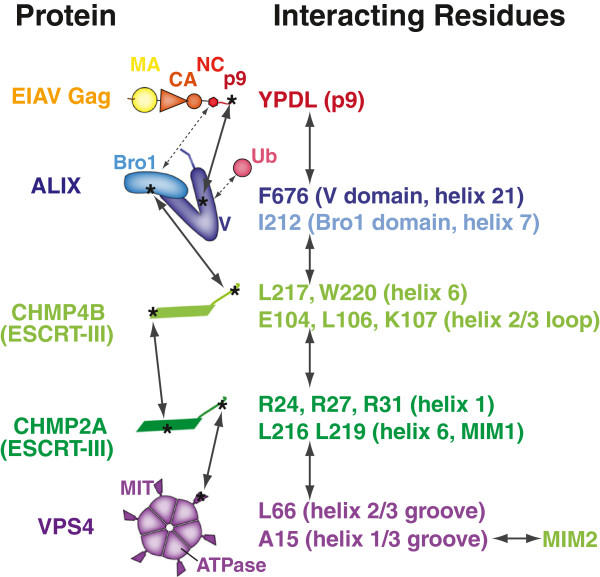
**Summary of ESCRT proteins and interactions that contribute to EIAV budding.** EIAV Gag, cellular ESCRT proteins, and their protein-protein interactions (solid arrows) shown here to be involved in EIAV budding are depicted schematically. ALIX interactions with ubiquitin (Ub) and NC^Gag^ interactions previously shown to contribute to virus release are depicted with dotted arrows. Specific residues whose mutation inhibit the different interactions are given at right, together with their location within the relevant protein.

Protein-protein interactions in the EIAV budding pathway are summarized in Figure 6. The YPDL late domain motif within EIAV p9^Gag^ binds ALIX [62], with the late domain tyrosine inserting into a hydrophobic pocket in the ALIX V domain [71]. ALIX can also bind calcium [81], dimerize [23], bind NC^Gag^ through the Bro1 domain [82-84], and bind Lys-63-linked polyubiquitin chains through the V domain [85,86], although these interactions have not yet been characterized structurally. ALIX, in turn, binds directly to CHMP4B via an interaction between a hydrophobic patch on the ALIX Bro1 domain and the C-terminal amphipathic helix of CHMP4B [87], and this interaction is required for EIAV budding (Figure 2).

ALIX appears to perform analogous “early” assembly functions in a series of other mammalian ESCRT-dependent processes, including abscission [88-90], MVB protein sorting [8], shedding microvesicle formation [91,92], and release of membrane-bound forms of “non-enveloped” viruses [93] and non-enveloped particles of “enveloped” viruses [94]. The use of ALIX to initiate ESCRT factor recruitment and assembly initially appeared to be an important difference between EIAV budding and MVB sorting in *S. cerevisiae*, where the apparent ALIX homolog, Bro1p, was reported to function *downstream* of Snf7p (CHMP4) [8]. A recent report indicates, however, that ALIX can also function early in yeast MVB biogenesis [95], and so the ESCRT recruiting activities of ALIX family members now appear to be widely conserved.

CHMP4B helps to recruit CHMP2A. CHMP4B and CHMP2A can interact directly *in vitro*[30,57], and a CHMP2A mutation that impairs CHMP4B binding *in vitro*[57] inhibited the ability of CHMP2A to function in EIAV budding (Figure 3B). A CHMP4B mutation that impairs CHMP2A binding *in vitro*[57] diminished, but did not eliminate EIAV release (Figure 3A). It is possible that these phenotypic effects were incomplete because these mutations do not block the CHMP4B-CHMP2A interaction completely and/or because CHMP3 helps bridge the CHMP4B-CHMP2A association [30,56]. A supporting role for CHMP3 in lentivirus budding is likely because CHMP3 binds tightly to both CHMP2 and CHMP4 proteins *in vitro*[30], appears to act together with CHMP2 proteins in HIV-1 budding, particularly CHMP2A [30], and performs an essential bridging role in an *in vitro* HIV-1-based ESCRT assembly system [56]. Nevertheless, our depletion experiments indicate that CHMP3 does not perform an absolutely essential role in EIAV budding under native conditions. Moreover, there are indications that CHMP2 and CHMP4 can function together in the absence of CHMP3 in other systems. For example, overexpression of Vps2p/Did4p (the CHMP2 homolog in *S. cerevisiae*) suppresses the temperature- and concavanine-sensitivity induced by deletion of Vps24p/Did3p (the CHMP3 homolog) from *S. cerevisiae*[96]. Similarly, eukaryotes like *P. falciparum* and *E. histolytica* appear to have CHMP4 and CHMP2 homologs, but lack CHMP3 homologs [97].

It is not yet clear why CHMP2A is the primary CHMP2 isoform required for EIAV release from 293T cells (Additional file 2: Figure S1A), nor why CHMP4B is the primary CHMP4 isoform required for release of EIAV (Additional file 2: Figure S1B) and HIV-1 [57,98,99]. CHMP4C, at least, is recruited to sites of EIAV assembly in HeLa cells [54], and the simplest explanation for our negative functional data is that the recruited CHMP4C proteins simply do not perform an essential (or non-redundant) role in virus budding. The CHMP2A:CHMP4B interaction is the only ESCRT protein:protein interaction shown in Figure 6 for which a structural model is not yet available, and this information will be important for revealing how ESCRT-III proteins co-polymerize and for identifying even more specific inhibitory mutations.

Like HIV-1, EIAV budding requires multiple VPS4 activities, including ATP, MIM1, and MIM2 binding (Figure 4 and ref. [34]). VPS4 is recruited to sites of EIAV and HIV-1 budding immediately prior to the fission step [54,55], and CHMP2A appears to be at least one important VPS4 partner because a CHMP2A mutant that lacked VPS4 binding activity failed to support EIAV budding (Figure 3B). The functional target(s) for VPS4B MIM2 binding activity is less clear. VPS4 proteins can bind CHMP4 proteins through MIM2-like interactions, but the isolated interactions are weak *in vitro*[34]. Nevertheless, the equivalent interaction between Snf7p (CHMP4) and Vps4p is functionally important for MVB protein sorting in yeast [100], so CHMP4-VPS4B interactions may also be functionally important during EIAV budding.

A significant new finding is that depletion of different ESCRT factors arrests EIAV budding at phenotypically distinct stages (Figure 5). CHMP4A/B depletion induced a particularly striking phenotype in which Gag processing was incomplete, and the virus formed multi-lobed virions and long tubes. We hypothesize that this is because CHMP4B recruitment normally helps activate Gag processing and/or inhibit Gag polymerization, which therefore continues unabated in the absence of CHMP4B. Similar tubular virions have been reported for mutant Moloney Murine Leukemia Viruses that carry deletions of the Gag p12 domain or the PPPY late domain [101,102]. HIV-1 Gag also overpolymerizes in budding-arrested virions, leading Kräusslich and colleagues to propose that ESCRT-mediated release occurs in kinetic competition with Gag polymerization [103]. These observations all indicate that, in addition to providing essential membrane fission activity, ESCRT factors can also help facilitate Gag processing and/or terminate Gag assembly. We previously reported that released HIV-1 Gag levels do not reliably correlate with infectivity reductions in cells lacking CHMP4A/B ([57] and see Figure 1A, lane 6). This observation suggests the possibility that analogous, but less dramatic Gag polymerization defects may also occur for HIV-1. Perhaps the extent of tubular virion formation is influenced by the predisposition of different retroviral Gag proteins to polymerize into spheres vs. tubes.

Unlike ALIX, which increases steadily throughout the process of Gag assembly, CHMP4B is recruited to sites of EIAV budding in short “bursts” that immediately precede virus budding [54]. Thus, there must be a “switch” (or switches) that activates the accumulating ALIX molecules, inhibits Gag polymerization, activates Gag processing, and recruits the late-acting ESCRT factors, CHMP4B, CHMP2A and VPS4. Factors that could trigger this switch include the proper degree of membrane curvature and/or critical concentrations of ALIX or Gag. Switch components could include ubiquitin transfer and/or conformational changes in Gag or ALIX. At that point, CHMP4B recruitment and polymerization in the bud neck could help block extension of the hexagonal Gag lattice. The switching process is likely to be complex, however, as suggested by the puzzling observation that tubular EIAV Gag overpolymerization phenotypes were not observed when ALIX was depleted (Figure 5), nor were elevated Gag release levels observed for ALIX mutants that lacked binding sites for CHMP4B (Figure 2) or ubiquitin [85].

## Conclusions

In summary, our experiments reveal that EIAV budding requires only a subset of ESCRT proteins, including ALIX, CHMP4B, CHMP2A and VPS4. Point mutations that inhibited the interactions between these proteins also inhibited their ability to function in EIAV budding, indicating that these proteins interact directly during the budding process. Long tubular virions are produced in the absence of CHMP4B, suggesting that the burst of recruitment of the late-acting ESCRT factors help mediate the switch from Gag polymerization to Gag processing and membrane fission. These studies help establish EIAV as a streamlined model system for dissecting the stepwise processes of lentivirus assembly and ESCRT-mediated budding.

## Methods

### Cell culture

293T and HeLa-TZM reporter cells were maintained in DMEM (Invitrogen) with 10% FCS. HeLa-TZM cells were obtained through the AIDS Research and Reference Reagent Program.

### siRNAs, expression vectors and antibodies

siRNA (19 nt + d(TT) overhangs) were designed using the Dharmacon siDESIGN Center (Thermo Fisher Scientific Inc.) and were synthesized by the University of Utah core facility. siRNA sequences are provided in Additional file 5: Table S1, expression vectors used in this study are provided in Additional file 6: Table S2, and most ESCRT antibodies and their working conditions are described in [104]. We raised our own rabbit anti-HIV CA (UT 416) and MA (UT 556) antisera (mixed together, each at 1:1,000 dilution), anti-EIAV CA (UT418, 1:3,000), anti-ALIX (UT 324, 1:500), and anti-EAP20 (UT461, 1:500). Murine anti-GAPDH (Millipore) was used at a dilution of 1:15,000.

### ESCRT protein depletion, rescue and EIAV virion production

Detailed protocols for siRNA depletion of the different ESCRT proteins, expression of exogenous, siRNA-resistant rescue constructs, and western blotting conditions are described in [57] (for equivalent HIV-1 experiments). Briefly, the experiments shown here in Figures 1, 2, 3, 4, and 5A, Additional file 2: Figure S1 and Additional file 3: Figure S2 were performed following the time course: t = 0, 293T cells seeded at 2 × 10^5^ cells/well in 6-well plates; t = 24 hr, transfection with 10 nM siRNA and 7.5 μl lipofectamine RNAimax (Invitrogen); t = 48 hr, media change (2 ml) and co-transfection with 10 nM siRNA, with control vector or siRNA-resistant expression construct (0.7 μg), and with control vector and viral expression vector(s) (0.5 μg HIV-1 R9 vector [105], or an EIAV vector system comprising 0.2 μg pEV53, 0.2 μg pSIN6.1CeGFPW and 0.075 μg phCMV-VSV-G [75,76] using 10 μl lipofectamine 2000 (Invitrogen); t = 72 hr media change (EIAV only); and t = 96 hr, harvest cells and culture supernatant for analysis. Western blots were used to analyze levels of released virion-associated viral proteins and soluble cellular proteins, with integrated band intensities measured with an Odyssey Imager, Li-Cor Biosciences. HIV-1 titers were analyzed on HeLa-TZM reporter cells. EIAV vector titers were determined by titrating culture supernatants onto 293T target cells and quantifying transduced cells expressing GFP 72 h post-infection by flow cytometry (FACScan, Becton Dickison).

### Transmission electron microscopy

Virus-producing 293T cells were pelleted at low speed, fixed with 2.5% glutaraldehyde/1% paraformaldehyde in cacodylate buffer (0.1 M sodium cacodylate (pH 7.4), 35 mM sucrose, 4 mM CaCl_2_) 48 h after the second siRNA transfection, washed three times for 10 min with 50 mM cacodylate buffer, stained with a 2% OsO_4_ solution for 1 h, rinsed three times for 10 min with water, and stained with a 4% uranyl acetate solution for 30 min. Samples were dehydrated with a graded ethanol series, then in acetone, and embedded in epoxy resin Embed-812 (Electron Microscopy Sciences). Thin sections (80–100 nm) were picked up on copper grids, stained for 20 min on drops of saturated uranyl acetate, rinsed with water and then stained for 10 min on drops of Reynolds’ lead citrate. Electron micrographs were collected on a Hitachi H-7100 transmission electron microscope at an accelerating voltage of 75 kV, equipped with a Gatan Orius sc1000 camera.

Two independent experiments were performed, and a total of 28 cells with associated EIAV virions were counted for each condition and scored for the presence of: mature virions, immature virions, budding virions (Gag assemblies with half-moon or “lollipop” morphologies) and multi-lobed or tubular virions (virions with multiple lobes or long tubular structures). Examples of these different phenotypes are given in Figure 5B, panel 4 and their relative percentages are provided in Figure 5C. ESCRT protein depletion generally increased the frequency of cells with observable cell-associated virions and the number of associated virions/cell. Thus, to find 28 cells with associated EIAV virions, we had to count a total of 1020 control cell sections, 1282 ALIX-depleted cell sections, 927 CHMP2A/B-depleted cell sections, and 428 CHMP4A/B-depleted cell sections. Scored virion numbers were: 198 (control cells), 365 (ALIX-depleted cells), 491 (CHMP2A/B-depleted cells) and 1172 CHMP4A/B-depleted cells).

## Competing interests

The authors declare that they have no competing interests.

## Authors’ contributions

VS performed the experiments, and VS and WIS analyzed the data and wrote the manuscript. Both authors read and approved the final manuscript.

## Supplementary Material

Additional file 1 Supplemental Text and References. Click here for file

Additional file 2: Figure S1 Effects of CHMP2 and CHMP4 depletion on EIAV release and infectivity. Click here for file

Additional file 3: Figure S2 Depletion of EAP20, CHMP3 or CHMP6 does not significantly affect EIAV release or infectivity. Click here for file

Additional file 4: Figure S3 Release of EIAV virions from control 293T cells. Click here for file

Additional file 5: Table S1 siRNA sequences used in this study. Click here for file

Additional file 6: Table S2 Expression vectors used in this study. Click here for file
