# Supplementary material for: ESCRT requirements for EIAV budding
Source: Retrovirology. 2013 Oct 9;10:104. doi: 10.1186/1742-4690-10-104 (PMC3907061; doi:10.1186/1742-4690-10-104)
Supplement: Additional file 1 — Supplemental Text and References. [file 1742-4690-10-104-S1.docx]

**Additional Material**

**ESCRT Requirements for EIAV Budding**

**Virginie Sandrin and Wesley I. Sundquist***

**Affiliations:** Department of Biochemistry, University of Utah School of Medicine, Salt Lake City, Utah 84112-5650, USA

*To whom correspondence should be addressed: Wesley I. Sundquist, Department of Biochemistry, University of Utah School of Medicine, Salt Lake City, UT, USA, Tel: (801) 585-5402; Fax (801) 581-7959; E-mail: [wes@biochem.utah.edu](mailto:wes@biochem.utah.edu)

Additional Material Included

1. Additional Results (Text)
2. Additional Tables (2)
3. Additional Figures and Captions (3)
4. Additional References (6)

**Additional Results**

Surveys of ESCRT Factors Required for EIAV Budding

siRNA depletion experiments were initially used to survey the dependence of EIAV budding on the mammalian homologs of the core yeast ESCRT factors, plus ALIX (Figure 1, Additional Figure 1 and Additional Figure 2). In these experiments, 293T cells were transfected with an EIAV expression vector, together with either control siRNAs or with siRNAs that individually targeted ALIX, TSG101 (ESCRT-I), EAP20 (ESCRT-II), CHMP2A, CHMP2B, CHMP3, CHMP4A, CHMP4B, CHMP4C, CHMP6 (all ESCRT-III) and VPS4A/B. We also tested the possibility that isoforms of CHMP2 (A and B) and CHMP4 (A, B and C) might function redundantly by testing the effects of co-depleting the different isoforms of CHMP2 and CHMP4 (Additional Figure 1A and 1B, respectively).

Viral titers were measured in single-cycle infectivity assays (panel 1), and virion release was analyzed using western blots to quantify the levels of virion-associated CA proteins released into the culture supernatant (panel 2, “Virus”). Western blots of soluble cell extracts were performed to visualize Gag protein expression and processing (panel 3), and the efficiency of target protein depletion (bottom panels). In every case except CHMP4C (where suitable antibodies were not available), the blots confirmed that siRNA treatment efficiently reduced target protein levels. Here, we present the results for the experiments that are not presented in the main text.

Roles of the Individual CHMP2 Proteins in EIAV Budding

CHMP2A appears to be the primary CHMP2 family member used for EIAV budding because depletion of CHMP2A alone reduced EIAV release and infectivity (Additional Figure 1A, lane 3, 28-fold reduction in release and 4-fold reduction in infectivity, albeit with a modest reduction in EIAV Gag expression), whereas depletion of CHMP2B alone had no significant effect on release or infectivity (lane 4). However, co-depletion of CHMP2B reduced EIAV infectivity even further than CHMP2A depletion alone (compare lane 5 to lanes 3 and 4, 17-fold reduction in release and 17-fold reduction in infectivity), indicating that CHMP2B can also contribute modestly to EIAV release and infectivity, at least when CHMP2A levels are low. The weaker reduction in EIAV Gag release shown here for co-depletion of CHMP2A and CHMP2B (vs. CHMP2A alone) was not reproducibly observed in other repetitions of this experiment. The greater infectivity reduction seen here for CHMP2A/B co-depletion was reproducible. Hence, CHMP2A and CHMP2B were co-depleted in all subsequent studies of the CHMP2 proteins.

Roles of the Individual CHMP4 Proteins in EIAV Budding

CHMP4B appears to be the primary CHMP4 family member used for EAIV budding because depletion of CHMP4B alone significantly reduced virus infectivity (Additional Figure 1B, compare lanes 1 and 4, 7-fold infectivity reduction). In contrast, CHMP4C depletion did not alter EIAV infectivity (compare lanes 1 and 5) and CHMP4A depletion reproducibly *increased* viral infectivity slightly (lane 3, 2-fold infectivity increase). In co-depletion experiments, CHMP4C also failed to synergize significantly with CHMP4A, CHMP4B, or both (lanes 7-9). We therefore conclude that CHMP4C does not contribute measurably to EIAV release. Co-depletion of CHMP4A and CHMP4B did reduce viral infectivity somewhat more than depletion of CHMP4B alone (Additional Figure 1B, compare lanes 6 and 4, 23-fold infectivity reduction). Hence, CHMP4B appears to be the primary CHMP4 member used in EIAV release, but CHMP4A may also contribute modestly. CHMP4A and CHMP4B were therefore co-depleted in all subsequent studies of the CHMP4 proteins

Depletion of EAP20, CHMP3 or CHMP6 Does Not Significantly Affect EIAV Release or Infectivity.

In addition to the results for CHMP2 and CHMP4 depletion described above, depletion of four other individual ESCRT proteins had no measurable effect on EIAV release or infectivity (TSG101, EAP20, CHMP3, and CHMP6). The effects of TSG101 depletion are reported in the main body of the paper because the comparison with HIV-1 is informative (compare lanes 4 from Figure 1A and 1B). In contrast, depletions of EAP20 (ESCRT-II), CHMP3 and CHMP6 had no effect on either EIAV or HIV-1 [[1](#_ENREF_1), [2](#_ENREF_2)], and the EIAV data are therefore simply reported here for completeness (Additional Figure 2). As shown in Additional Figure 2, siRNA treatments depleted EAP20 and CHMP6 efficiently (bottom panels, 8-fold depletion in each case), but had no significant effects on EIAV release, infectivity, or cellular Gag expression (panels 1-3, compare lanes 4 and 5 to lanes 1 and 2). These results are consistent with the observed TSG101 (ESCRT-I) independence of EIAV because ESCRT-II binds and acts downstream of ESCRT-I, and CHMP6 (ESCRT-III) binds and acts downstream of EAP20 (ESCRT-II).

In *S. cerevisiae*, Vps24p (CHMP3) performs an essential role in MVB sorting, where it acts to bridge the ESCRT-III subunits, Vps32p/Snf7p (CHMP4) and Vps2p/Did4p (CHMP2) [[3](#_ENREF_3)]. The role of CHMP3 in retrovirus budding has been less clear because CHMP3 depletion alone does not inhibit HIV-1 release significantly [[1](#_ENREF_1)], but CHMP3 depletion can synergize with CHMP2 depletion [[4](#_ENREF_4)]. We have therefore concluded that CHMP3 is not essential for HIV-1 budding, but can contribute to the process, presumably by helping to bridge CHMP2 and CHMP4 proteins. A bridging role for CHMP3 is also consistent with the observations that: 1) CHMP3 can bind both CHMP2A and CHMP2B in vitro [[4](#_ENREF_4)], 2) although CHMP2A and CHMP2B can bind CHMP4B *in vitro*, CHMP3 binds CHMP4B with higher affinity than does CHMP2A [[4](#_ENREF_4)], and 3) an analysis of human ESCRT-III protein recruitment to membranes *in vitro* concluded that CHMP3 is required for efficient CHMP2 recruitment [[5](#_ENREF_5)].

As shown in Additional Figure 2B, we identified two different siRNA oligouncleotides that depleted CHMP3 to nearly undetectable levels (bottom panels, >50-fold depletion). Although one of these siRNA oligonucleotides (CHMP3-474) diminished EIAV Gag expression slightly, neither had a significant effect on EIAV release or infectivity (compare lanes 3 and 4 to lane 1, >3-fold effects on virus release and infectivity). Thus, although we cannot rule out the possibility that residual CHMP3 protein levels are sufficient to support EIAV release, we favor the idea that CHMP3 plays a non-essential, but possibly supporting role in EIAV budding.

**Additional Figure Captions**

**Additional Figure 1. Effects of CHMP2 and CHMP4 depletion on EIAV release and infectivity.** Viral titers (top panel), and western blots showing levels of virion-associated Gag proteins (panel 2, “Virus”) and the designated intracellular viral and cellular proteins in 293T cells expressing EIAV (lower panels, “Cell”). (**A**) EIAV-producing cells were treated with a control siRNA (lane 1) or siRNAs that depleted ALIX (positive control, lane 2), CHMP2A (lane 3), CHMP2B (lane 4) or both CHMP2A and CHMP2B (lane 5). Bottom panels show cellular levels of GAPDH (loading control) and the designated CHMP2 protein. Integrated band intensities are shown for virus release (panel 2, CA band intensities) and target protein depletion (bottom panels), and values are relative to the control lane. (**B**) EIAV-producing cells were treated with a control siRNA (lane 1) or siRNAs that depleted ALIX (positive control, lane 2), CHMP4A (lane 3), CHMP4B (lane 4), CHMP4C (lane 5), both CHMP4A and CHMP4B (lane 6), both CHMP4A and CHMP4C (lane 7), both CHMP4B and CHMP4C (lane 8), or CHMP4A, CHMP4B and CHMP4C (lane 9). Bottom panels show cellular levels of GAPDH (loading control) and the designated CHMP4 protein (note that a sui antibody was not available for CHMP4C, but this siRNA has been shown to deplete exogenously expressed CHMP4C [[6](#_ENREF_6)]). Band intensities were integrated for the shown blots to quantify levels of virus release (panel 2, CA band intensities) and target protein depletion (bottom panels), and values are relative to the control lane. Error bars in the top panel show the range from the mean of two independent repetitions of the experiment, performed in parallel.

**Additional Figure 2. Depletion of EAP20, CHMP3 or CHMP6 does not significantly affect EIAV release or infectivity.** Viral titers (top panel), and western blots showing levels of virion-associated Gag proteins (panel 2, “Virus”) and the designated intracellular viral and cellular proteins in 293T cells expressing EIAV (lower panels, “Cell”). (**A**) EIAV-producing cells were treated with control siRNAs (lanes 1 and 2) or siRNAs that depleted CHMP2A and CHMP2B (positive control, lane 3), EAP20 (lane 4) or CHMP6 (lane 5). Bottom panels show cellular levels of the designated ESCRT protein following treatment with the control siRNA (equivalent to control lanes 1 and 2) or a specific siRNA (equivalent to the lane designated beneath the blot). Band intensities were integrated for the shown blots to quantify levels of virus release (panel 2, CA band intensities) and target protein depletion (bottom panels), and values are relative to the average intensities of the two control lanes. (**B**) Virus-producing cells were treated with a control siRNA (lane 1) or siRNAs that depleted ALIX (positive control, lane 2), or two different siRNA duplexes that target CHMP3 (lanes 3 and 4, number denotes the starting nucleotide of the targeted site in the CHMP3 mRNA coding region). Bottom panel shows cellular levels of CHMP3 following the different siRNA treatments. Integrated band intensities are shown for virus release (panel 2, CA band intensities) and target CHMP3 depletion (bottom panel), and values are relative to the control lane. Error bars in the top panel show the range from the mean of two independent repetitions of the experiment, performed in parallel.

**Additional Figure 3. Release of EIAV virions from control 293T cells.** Thin section transmission EM images showing cell-associated EIAV virions from wild type 293T cells (control for images shown in Figure 5B). Scale bar is 100 nm.

**References**

1. Morita E, Sandrin V, McCullough J, Katsuyama A, Baci Hamilton I, Sundquist WI: **ESCRT-III Protein Requirements for HIV-1 Budding.** *Cell Host Microbe* 2011, **9:**235-242.

2. Langelier C, von Schwedler UK, Fisher RD, De Domenico I, White PL, Hill CP, Kaplan J, Ward D, Sundquist WI: **Human ESCRT-II complex and its role in human immunodeficiency virus type 1 release.** *Journal of virology* 2006, **80:**9465-9480.

3. Babst M, Katzmann D, Estepa-Sabal E, Meerloo T, Emr S: **Escrt-III. An endosome-associated heterooligomeric protein complex required for mvb sorting.** *Dev Cell* 2002, **3:**271-282.

4. Effantin G, Dordor A, Sandrin V, Martinelli N, Sundquist WI, Schoehn G, Weissenhorn W: **ESCRT-III CHMP2A and CHMP3 form variable helical polymers in vitro and act synergistically during HIV-1 budding.** *Cellular microbiology* 2013, **15:**213-226.

5. Carlson LA, Hurley JH: **In vitro reconstitution of the ordered assembly of the endosomal sorting complex required for transport at membrane-bound HIV-1 Gag clusters.** *Proc Natl Acad Sci USA* 2012, **109:**16928-16933.

6. Morita E, Colf LA, Karren MA, Sandrin V, Rodesch CK, Sundquist WI: **Human ESCRT-III and VPS4 proteins are required for centrosome and spindle maintenance.** *Proc Natl Acad Sci USA* 2010, **107:**12889-12894.
