# Supplementary figures and images for: ESCRT requirements for EIAV budding
Source: Retrovirology. 2013 Oct 9;10:104. doi: 10.1186/1742-4690-10-104 (PMC3907061; doi:10.1186/1742-4690-10-104)

FIGURE S1

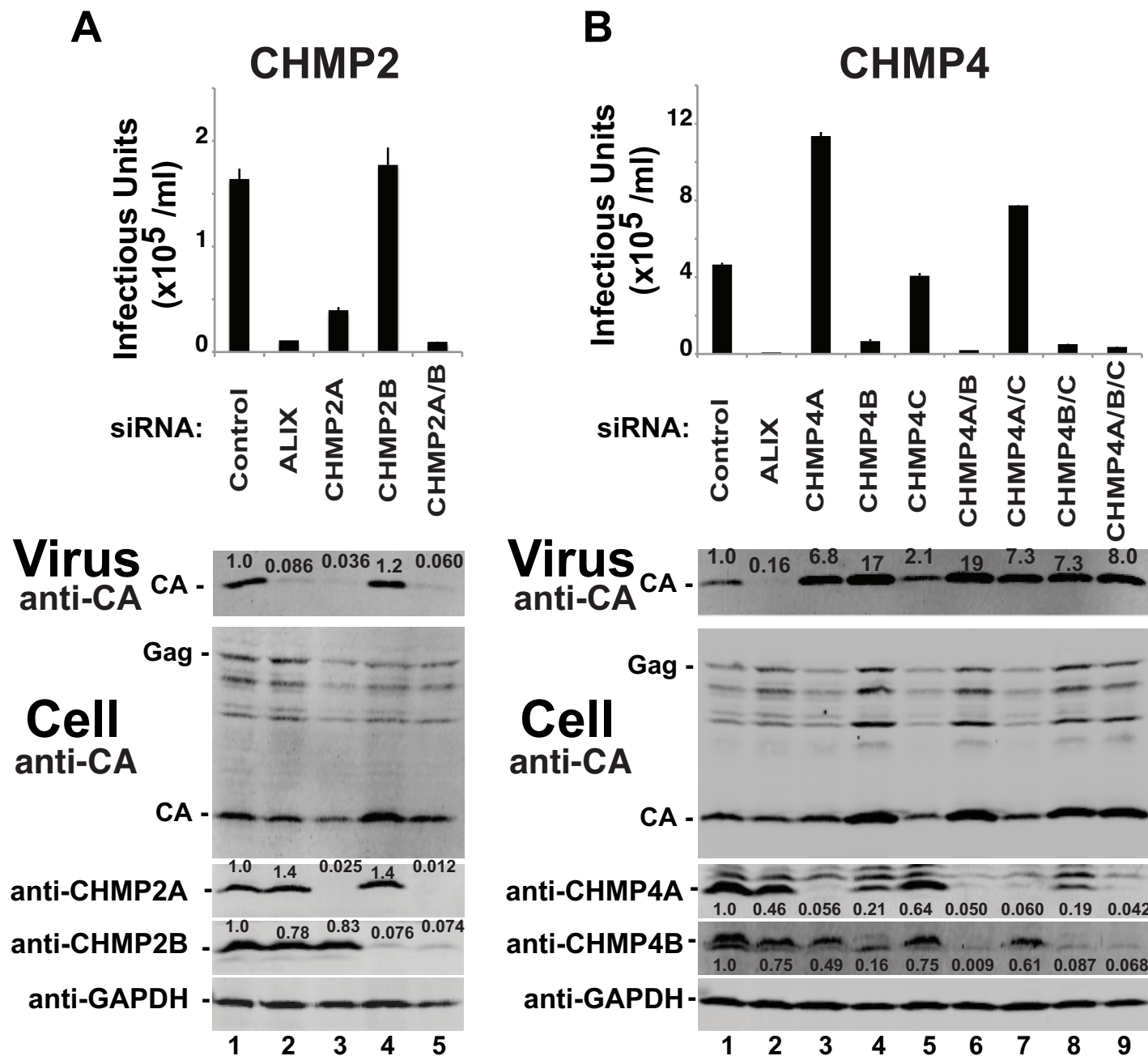

Supplement: Additional file 2: Figure S1 — Effects of CHMP2 and CHMP4 depletion on EIAV release and infectivity. [file 1742-4690-10-104-S2.pdf]

FIGURE S2

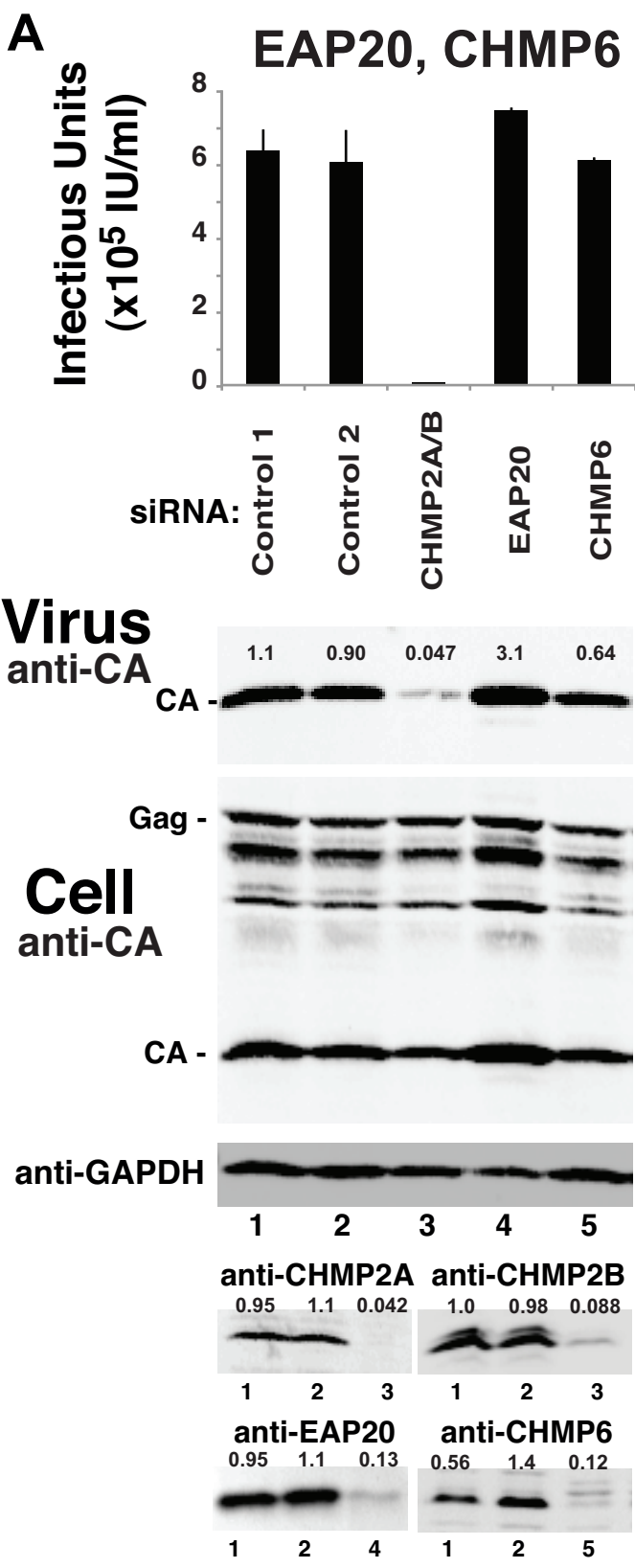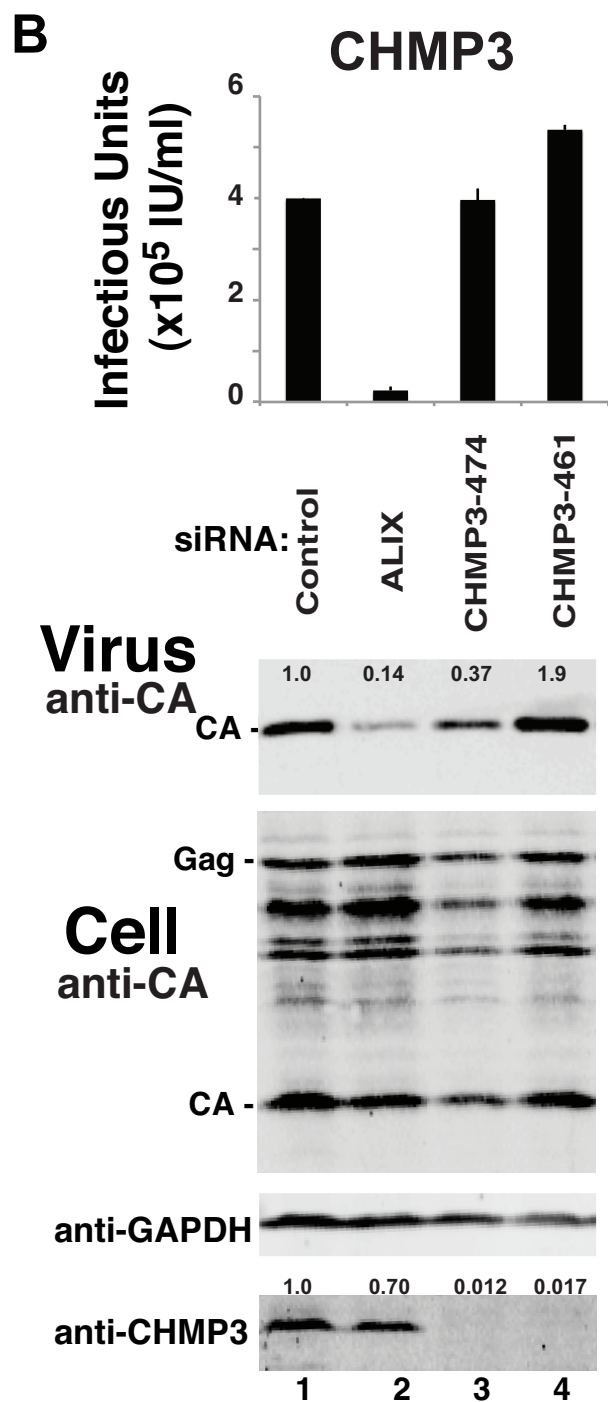

Supplement: Additional file 3: Figure S2 — Depletion of EAP20, CHMP3 or CHMP6 does not significantly affect EIAV release or infectivity. [file 1742-4690-10-104-S3.pdf]

**Figure S3**

**Control**

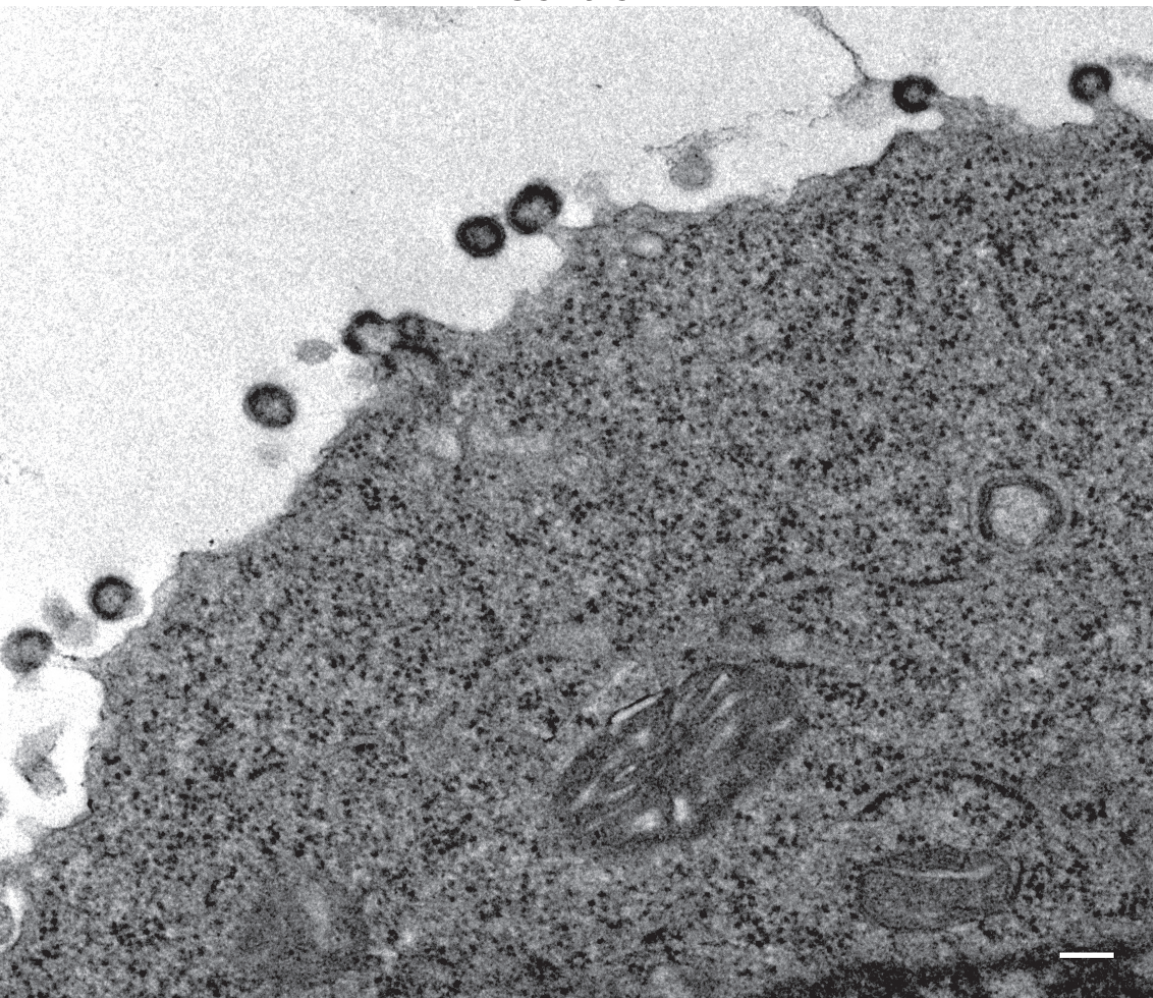

Supplement: Additional file 4: Figure S3 — Release of EIAV virions from control 293T cells. [file 1742-4690-10-104-S4.pdf]
