# Supplementary material for: ESCRT requirements for EIAV budding
Source: Retrovirology. 2013 Oct 9;10:104. doi: 10.1186/1742-4690-10-104 (PMC3907061; doi:10.1186/1742-4690-10-104)
Supplement: Additional file 6: Table S2 — Expression vectors used in this study. [file 1742-4690-10-104-S6.docx]

**TABLE S2: Expression vectors used in this study**

| **Plasmid Name** | **Internal ID** | **Cloning sites** | **Mutations** | Source* |
| --- | --- | --- | --- | --- |
| **Virus production vectors** |  |  |  |  |
| R9 | MTA (WISP96-96) |  |  | Swingler et al., 1997 |
| pEV53  (EIAV structural proteins) | MTA |  |  | Olsen, 1998 |
| pSIN6.1CeGFPW  (packagable GFP expression vector) | MTA |  |  | Olsen, 1998 |
| phCMV- VSV-G  (envelope) |  |  |  | Yee et al., 1994 |
| **Mammalian rescue expression vectors** |  |  |  |  |
| pCMVΔ3MCS2 | WISP12-94 |  |  | Morita et al., 2012 |
| pCMVΔ3-ALIX | WISP12-86 |  |  |  |
| pCMVΔ3-ALIX _C4-_ | WISP12-87 |  | I212D |  |
| pCMVdelta4.5MCS2 | WISP09-144 |  |  |  |
| pCMVΔ4.5-CHMP2A | WISP09-147 | KpnI, XhoI |  | NP_055268 |
| pCMVΔ4.5-CHMP2A_VPS4-_ | WISP10-649 | Quikchange Mutagenesis | L216D L219D | Morita et al., 2011 |
| pCMVΔ4.5-CHMP2A_CHMP4-_ | WISP10-296 | Quikchange Mutagenesis | R24A  R27A  R31A | Morita et al., 2011 |
| pCMVΔ4.5-CHMP4B | WISP09-152 | KpnI, XhoI |  | NP_789782 |
| pCMVΔ4.5-CHMP4B_ALIX-_ | WISP09-154 | Quikchange Mutagenesis | L217A W220A | Morita et al., 2011 |
| pCMVΔ4.5-CHMP4B_CHMP2-_ | WISP10-270 | Quikchange Mutagenesis | E104A V105A L106A K107A | Morita et al., 2011 |
| pcDNA3.1-VPS4B | WISP08-64 | EcoRI/BamHI |  | AF038960 |
| pcDNA3.1-VPS4B_ATPase-_ | WISP08-65 | Quikchange Mutagenesis | K180Q | Kieffer et al., 2008 |
| pcDNA3.1-VPS4B_MIM1-_ | WISP08-66 | Quikchange Mutagenesis | L66D | Kieffer et al., 2008 |
| pcDNA3.1-VPS4B_MIM2-_ | WISP08-67 | Quikchange Mutagenesis | A15D | Kieffer et al., 2008 |
| pcDNA3.1-VPS4B_MIM1/2-_ | WISP08-68 | Quikchange Mutagenesis | L66D, A15D | Kieffer et al., 2008 |

* “Source” corresponds to the NCBI protein accession number or the ATCC numbers or the paper describing these constructs.
